# Sustainability of evidence-based primary care programs in Abu Dhabi: impact of COVID-19

**DOI:** 10.3389/frhs.2025.1591667

**Published:** 2025-08-25

**Authors:** Latifa Baynouna Alketbi, Nico Nagelkerke, Hanan Abdelbaki

**Affiliations:** ^1^ Abu Dhabi Healthcare Services, Academic Affairs, SEHA Clinics, AlAin, United Arab Emirates; ^2^ College of Medicine and Health Science, United Arab Emirates University, AlAin, United Arab Emirates

**Keywords:** sustainability, evidence-based intervention, primary care, Abu Dhabi, COVID-19, ambulatory healthcare services, patient-centered medical home, chronic disease care

## Abstract

**Introduction:**

The Abu Dhabi Ambulatory Healthcare Services (AHS) implemented the Chronic Disease Care (CDC) and Patient-Centered Medical Home (PCMH) programs. This study describes the implementation and integration of these two programs into the operations of AHS centers and assesses their long-term sustainability.

**Method:**

This retrospective cohort study included all AHS centers. The sustainability of both programs was assessed twice yearly using the NCQA self-assessment audit and the CDC system audit. The care outcomes were measured through routinely collected Key Performance Indicators (KPIs) at multiple data collection points in 2018, 2021, and 2022. The study period included the COVID-19 period, providing an opportunity to examine the effect of pre-pandemic program scores on centers' performance during the pandemic. The KPIs are categorized into clinical, preventive, and utilization outcomes.

**Results:**

Linear regression showed that the key performance indicator (KPI) for the best-performing centers had significantly higher PCMH scores, *B* = 0.447, *p* = 0.03, with no effect on the centers' financial revenue, *B* = 0.209, *p* = 0.29. Similarly, using univariate linear regression, a higher chronic disease care program assessment at the end of 2022 was significantly associated with centers performing better in clinical KPI, *B* = 0.480, *p* = 0.013. The Chronic Diseases Program assessment at the end of 2022 was as well positively and significantly associated with higher performance of centers regarding the NCQA PCMH standards, *B* = 0.647, *p* < 0.001. Pearson correlation analysis indicated significant correlations between clinical and preventive KPI achievements and the 2022 PCMH and CDC scores.

**Conclusion:**

The AHS centers successfully implemented both programs sustainably. The study findings highlight areas for sustainability research that demonstrate the value of sustainable interventions.

## Contributions to the literature

• Deviations in achieving optimal healthcare outcomes are rooted in the lack of enough evidence-based interventions.
• Evidence-based interventions, like providing family medicine-based primary care, rank among the most thoroughly studied interventions.
• This study demonstrates the sustainability of the well-known, evidence-based intervention, NCQA PCMH standards.
• The established structure and processes for adapting the NCQA PCMH standards supported AHS centers during and after the COVID-19 pandemic, resulting in superior clinical and utilization outcomes in centers that implemented the standards more effectively.

## Key messages

• Regulators should require standards based on the best evidence, such as those from the PCMH and CDC, to be integrated into the operations of primary healthcare centers. Deviations from these standards in areas such as population health management, care coordination, continuity, and disease management result in decreased quality, safety, and efficiency in patient care.
• Supporting practice improvement personnel, such as facilitators, care coordinators, and experts in practice improvement, is crucial. There must be a budget allocation for building capacity in these areas and for implementing their roles.
• Leadership and authoritative support, provided through built-in structures for continuous practice improvement, are crucial for the sustainability and effectiveness of PCMH and CDC standards implementation. Regulatory requirements should include not only standards enforcement but also sustained governance and monitoring systems within the organization or practice to ensure improved outcomes, program continuity, and sustainable implementation.
• Academic institutions and organizations should facilitate patient access to physicians with the skills and training to deliver whole-person, comprehensive, and longitudinal care by offering policy recommendations on professionalism, payment models, training, licensure, research, and developing undergraduate and postgraduate curricula in these areas (1).

## 1 Background

Studies on healthcare services are neither designed nor funded for sufficiently long periods to examine adaptation, sustainability, or outcomes of healthcare changes or interventions over time (2). Thus, implementation science requires later-stage translation research questions for population impact. It is important to prioritize the current evidence gap in this area by focusing on the value of sustaining interventions over time, identifying sustainability correlations and strategies for sustaining evidence-based interventions. and advancing workforce capacity, research culture, and funding mechanisms (3).

However, definitional issues regarding sustainability remain (4). Scheirer and Dearing define sustainability for program elements as the continued use of components and activities to maintain desirable program and population outcomes. This highlights the importance of continuous delivery and realizing benefits over time. Scheirer's work emphasizes that sustainability encompasses more than just continuing activities; it involves preserving benefits, adapting the program, and ensuring delivery capacity (5). Additionally, sustainability is seen as not only centered on whether an improvement program continues to exist, but also on whether normalization has been adopted. In this view, “new ways of working and improved outcomes become the norm” without reverting to previous practices and maintaining better outcomes, which is critical in defining sustainability (6).

Many countries devote significant effort to the spread and scale-up of healthcare service improvements; however, a few of those that succeed locally are spread and sustained widely (6). Another challenge in sustainability research is the ongoing change in the context and healthcare systems, increasing complexity of healthcare, resource constraints, growing diversity of populations, and the highly variable healthcare structure and process. Thus, healthcare leaders and managers rely on expertise to inform their decisions and, in many instances, for short-term gains mostly arising from problem situations (7). Thus, developing and implementing cost-effective healthcare services without sustainability has contributed to the availability of evidence-based guidelines, standards, and policies that can improve healthcare, but without sufficient evidence of their sustainability. This dearth in sustainability research prompts epistemological and methodological questions (8, 9).

This study describes an experience of sustaining healthcare programs in a new context and country. Abu Dhabi Ambulatory Healthcare Services (AHS) sustained the implementation of two best evidence-based standards for the provision of primary care for over a decade. They are the Chronic Disease Care (CDC) program, based on the chronic care model (CCM) by Wagner, implemented in 2004, and the Patient-Centered Medical Home (PCMH) program by the National Commission for Quality Assurance (NCQA), implemented in 2013 (10, 11). The goals of both programs align and overlap in terms of time and shared components. This provides additional learning potential for practice improvement through multiple solutions with changing contexts and challenges. Given that Abu Dhabi's healthcare system has been fee-for-service and open-access since 2008, it offers a distinct case study for implementing and sustaining PCMH/CDC, with its own set of challenges and solutions worth highlighting. Both programs have been reported in previous publications (12, 13), and their impact has been reviewed (14).

The CCM, a chronic disease management program, was conceptualized and implemented in primary care in 1998. The most known framework is the CCM by Wagner, who summarized the best research evidence in the CCM to guide quality improvement. The effectiveness of this model was evident in processes, health services, quality of life (QoL), health outcomes, satisfaction, and costs, in addition to an observed decrease in coronary heart disease mortality (11, 15). In a systematic review of 77 papers on the effectiveness of the CCM, all but two reported improvements in healthcare practice or health outcomes for people with chronic diseases. The systematic review revealed hidden determinants (16), including leadership commitment, awareness of end-users and reflective healthcare practice, and leaders' support of the implementation and sustainability of interventions, which were just as important as the CCM elements.

The NCQA program (10) has six well-established best-evidence standards. It covers service provision in primary care based on family medicine principles. The standards are focused on team-based care, knowing and managing populations, access and continuity, care management, care coordination, and performance measurement. Its effectiveness in delivering high-value care has been reported (12, 17–26).

This study examines the long-term sustainability of the CCM-based CDC and NCQA PCMH programs. Additionally, it tests the hypothesis that centers with higher PCMH/CDC scores pre-pandemic will show faster post-COVID recovery. The successful effects of these programs on patient outcomes developed in the US and their transferability can offer insight for generalizability.

## 2 Method

### 2.1 Study design

A retrospective cohort study of all AHS centers. There were multiple data collection points in 2018, 2021, and 2022, aiming to examine the sustainability of both programs. The effectiveness of both programs was studied during the initial implementation stage using an experimental design involving control centers, as reported in a previous publication. In this study, the emphasis was on the sustainability of the programs and their impact on care outcomes during the COVID-19 pandemic. This study was included all AHS centers from 2018 to 2023. During this period there was minimal leadership change and staff turnover.

### 2.2 Setting

Abu Dhabi citizens register with AHS in primary healthcare centers. Comprehensive healthcare services in family medicine include urgent, chronic, and preventive services, as well as specialty services, such as pediatrics, obstetrics, and gynecology. The centers are equipped with in-center imaging, x-ray and ultrasound, pharmacy, laboratory, and dental facilities. The payment system is an open-access fee for services covered by the government for United Arab Emirates (UAE) nationals. Non-UAE nationals or their employers cover health insurance plans. Family physicians, general practitioners, and specialists provide care. The population and healthcare system of Abu Dhabi have been described (14, 27, 28).

### 2.3 History of the programs

#### 2.3.1 Chronic disease care program

The CDC program was established in 2004. It boasted a system change in the centers in terms of structured daily clinics for patients with chronic diseases and a fixed schedule of the trained healthcare team led by a physician. In addition, EMR was continuously optimized to meet the needs of patients. Biannually, a comprehensive audit is conducted to benchmark the centers against each other according to the standards implemented.

#### 2.3.2 Patient-centered medical home program

Based on NCQA PCMH standards and the self-assessment that identified AHS gaps toward meeting the standards, a road map with multiple initiatives was developed. For each AHS department, medical, nursing, patient experience, pharmacy, dental, school health, health informatics, registration, allied health, and quality tasks were communicated and followed. The progress and approval of changes were tracked by a steering committee in the medical office for PCMH issues. Progress was based on the six NCQA standards and their updates and extended to areas targeted because of the local settings and unique opportunities for improvements. An example of modification is the requirement of a Primary Care Dentist (PCD) and the development of a population and panel management report from the EMR. Similarly, each school had a primary school nurse, an empanelment was performed, and reports were extracted for the panel management of school nurse students. Elements in the PCMH are presented in Supplementary Appendix 2.

### 2.4 Data collection

This study used routinely gathered organizational KPIs for the CDC and PCMH. The KPI are categorized as clinical and preventive KPI, Supplementary Appendix 3, and are monthly extracted and published from built EMR reports. All KPI are developed, validated, and approved by the quality department. The quality department publishes the KPI both in graphical form and as raw data for use in improving any identified gaps. Both programs assessed and tracked many outcomes. To simplify the analysis, the results were presented as measures of adherence to both programs (the accumulative annual score of the PCMH assessment and the CDC annual assessment score). Key Performance Measures related to care outcomes were grouped into preventive, clinical, utilization, and processes.

Most of the preventive and clinical KPIs were published monthly by the quality department. They included metrics on diabetes mellitus, cancer screening preventive programs, hypertension, depression and anxiety screening, and asthma. The central implementation committee developed additional clinical KPIs to monitor the newly implemented population health and disease Management programs were also published monthly. These included CKD, heart and stroke, smoking cessation, prediabetes, dyslipidemia, complex patients, and obesity. Other KPI were related to the program implementation process, such as medication reconciliation, referral feedback, investigation tracking, message center utilization, use of point-of-care decision support, risk stratification rate, departmental summary generation, primary dentist assignment, and PCP empanelment. Utilization KPIs included calling patients who were admitted or visited the emergency room, panel management by the PCP, and panel size adjustment. The benchmarking for better performance was based on international references, such as the NCQA recognition programs or the organizational KPIs, if international benchmarking was not used by the organization, such as for depression screening.

Two major audits were conducted biannually to assess compliance with the CDC and PCMH programs in May–June (mid-year) and December–January (end-of-year). The PCMH NCQA self-assessment audit is a score of the accumulation of points gained by the center for meeting elements in the six PCMH standards. For example, the Care Management and Support (CM) standard has two competencies, A and B weighing 10 points of the total score (four core, all must be met, and six electives, few can be not met). Competency A weighed six points for three elements to be met, two points for two core elements, and two points for one elective element. Supplementary Appendix 2A lists all point distributions; all points were added together with the AHS centers required to achieve 97 points. Although the total points required by the NCQA tool were 123, the AHS program required 97, as there were elements in the program that were still in progress of development by the Practice Improvement Committee, or it was not yet available in the AHS. Examples are two-way electronic communication between patients and healthcare providers, systematically obtaining prescription claims data to assess and address medication adherence, and publicly reporting clinician performance. The audit is performed by the program facilitators, and evidence is required for each element, which renders the assessment more objective. Further efforts to minimize potential bias in assessment included the program facilitator conducting physical site visits and collecting field evidence of adherence to the standards. As well, the care coordination facilitator extracts EMR reports on various care coordination activities and centers' performance with regard to the PCMH standards. Both facilitators compile the final self-assessment NCQA audit and discuss any disagreements with the project leadership. There are no subjective entries used in the audit.

Sustainability was assessed over the years with the persistence of adherence to the elements of the standards and evidence provided to earn score points. The present NCQA self-assessment was based on the 2017 NCQA PCMH standards, although an older version based on the 2011 and 2014 NCQA standards was previously used. The CDC program employed an internally built audit tool (Supplementary Appendix 2B) based on earning points for adherence to the elements that were developed over the years by the Practice Improvement Committee and as well evidence is required. The results of both audits are distributed to all centers for action plans and benchmarking.

The data and evidence were EMR extracts, audits from EMR reviews, or field verification by the auditor. Very few elements were manually audited, e.g., care plan documentation. Centers receive the results and can provide feedback on the data or KPI validity as they have the raw data, and they can identify patients with gaps and verify the accuracy of such gaps.

### 2.5 Interventions details

#### 2.5.1 Governance structure

In Abu Dhabi, healthcare facilities are governed by a regulatory body, the Department of Health (DOH), and healthcare service centers and hospitals are the operators. The AHS network of primary healthcare centers is linked to six government hospitals for secondary care. For patient care, AHS and all hospitals are linked with one Electronic Medical Record (EMR) introduced in 2009. The DOH publishes policies and screening and management guidelines. It oversees the implementation of national health programs and any cost coverage from the government. In addition, AHS implements certain programs as practice improvement initiatives (Supplementary Appendix 1).

PCMH and CDC programs have been overseen by the Academic Department through the Practice Improvement Committee since 2004. The Committee has been reporting to the PCMH steering committee since 2013.

#### 2.5.2 Communication and training

Communication with the healthcare centers occurs through facilitators in regular practice improvement meetings where key AHS staff are invited. At least once annually, a collaborative meeting is conducted to share the experiences and success of the center teams in CDC and PCMH implementation. An annual PCMH conference is conducted with experts in PCMH from the United States. Organizational training is provided for the center teams through the Continuous Professional Department in Academic Affairs.

#### 2.5.3 Program facilitation

The project lead and two full-time facilitators (practice improvement and care coordination facilitators) were responsible for facilitating and monitoring the program. The practice improvement facilitator is responsible for the agenda for implementation. She conducts site visits to all centers at least four times a year and communications through calls, virtual meetings, and emails. The central practice improvement committee approves an agenda, which the practice improvement facilitator delivers to the centers during a prearranged site visit. The visit can be geared toward training and informing the center teams of new changes or projects or auditing to ensure adherence to standards. The main agenda of the facilitation visits is a new addition to the program. Many ideas come from the center teams, and if suitable, are disseminated after approval by the practice improvement committee.

#### 2.5.4 Care coordination

The care coordination facilitator is responsible for care coordination elements in the NCQA PCMH standards, including empanelment, population health programs, core coordination care management, and PCMH metrics. She works on the EMR by coordinating its building, validation, extraction, and dissemination. Each center must have one care coordinator. She trains care coordinators and supports their ongoing queries. She audits their work and implements care coordination tasks for patients through the following changes and implemented strategies:

1. Maintaining the patient panel of the primary care physician (PCP) and continuous cleanup of such panels.
2. Population health lists are provided to the centers monthly to coordinate care and management. The programs implemented are Chronic Kidney Diseases, Ischemic Heart Diseases, Stroke, Osteoporosis, Dyslipidemia, Undiagnosed Hypertension, and Prediabetes.
3. Tracking the use of EMR reports and safety initiatives, such as investigations, referrals, and medication safety KPIs.
4. Program-specific metrics are prepared by the care coordination facilitator and published to all centers monthly, in addition to the KPI of the institution.

#### 2.5.5 Use of electronic medical records and program metrics

Both programs were facilitated by different supporting factors. First, the unified integrated Health Information System with the accumulative patient information data is a tool used to build and generate reports to identify different populations. It is the main method for identifying and tagging populations. Second, patient stratification based on published strategies (29) was implemented over the years based on either disease, population encounter, or global risk assessment. The disease stratification was based on diabetes, hypertension, asthma, prediabetes, ischemic heart disease, stroke, or chronic kidney disease (CKD). Another supporting factor was the national government programs with patients registering for preventive services, such as premarital, well-childcare, school screening, cardiovascular screening programs, or cancer screening, which increased patient engagement with the centers.

A global risk assessment was performed by the PCP clinical institution based on a modified American Academy of Family Physicians (AAFP) risk stratification (30). The stratified groups of patients were recalled and booked for appointments with the PCP for proactive care, disease management, and the identification of uncontrolled high-risk patients in their panel. EMR reports were used to monitor implementation, and feedback to the AHS centers and PCP is sent monthly.

#### 2.5.6 Awareness program

The PCMH program was identified as “Baytona Altebi”, meaning “Our Medical Home”. A continuous awareness program began since the launch of the PCMH program in 2013 for AHS employees and patients under this name. The launch of the employee program was through the first PCMH conference attended by senior leadership and officially announced as a government-supported initiative. Speakers with depth expertise in PCMH were invited. The community awareness is continuous, accompanied by logos, and placed on the buildings of all centers and educational material. Educational materials were developed, and the patients received PCP letters to introduce them to the PCP concept, accountability, and continuity. The CDC program was identified by this abbreviation, and within the EMR system, care was structured under this name.

### 2.6 Statistical analysis

KPI and audit data were summarized, graphed, analyzed, and reported using Microsoft Excel® (Microsoft, Redmond, WA) and SPSS Version 29. Descriptive statistics, frequency, and the percentage of adherence to PCMH and CDC standards were calculated. Univariate and multivariate linear regressions were employed to assess the association between the performance outcomes related to the KPI and the PCMH and CDC scores. The multivariable model was adjusted for variables such as financial performance, city, and being a rural center. Pearson correlation analysis (PCA) was employed to assess the correlation of PCMH and CDC scores with other important variables, such as the centers' financial revenue, laboratory imaging, and pharmacy orders, and a linear mixed-effects model was used to account for center-level clustering. Statistical significance was set at *p* < 0.05.

### 2.7 Ethics approval and consent to participate

The study was approved by the DOH-Abu Dhabi Institutional Review Board (IRB). All the methods were conducted following relevant guidelines and regulations. Informed consent was waived by the DOH-Abu Dhabi IRB, as the study was designed for retrospective data gathered as part of a patient care and organization quality program and anonymized during analysis.

## 3 Results

### 3.1 Population description

In AHS centers, females were more frequent attendees, 60.9% compared to 39.1% among males. Children under 18 years constitute 42.6%, compared to 46.5% in the age group between 18 and 59 years. Patients aged 60 years or older account for 10.9% of the attendees. Mainly, UAE nationals were the main visitors to the centers. The average centers' financial revenue in 2021 and 2022 was mainly from consultation visits (79.9%), preventive visits (15.8%), and teleconsultations (4.2%). It varied by the sizes of the centers, the 26 centers included seven rural centers, which had a considerably low number of patients.

### 3.2 Program adherence

Most of the 26 centers implemented both programs well, with excellent sustainability and progress over time. Table 1 shows a drop in the PCMH scores and KPIs during the COVID-19 pandemic. Furthermore, Table 1 shows the CDC program adherence score and the score of the NCQA self-assessment audit in 2018, 2021, and 2022.

**Table 1:** Description of the assessment variables used in studying the value and sustainability of the NCQA PCMH and the chronic disease care standards in the 26 centers.

	No. of centers	Minimum	Maximum	Mean (95% CI for mean)	SD
PCMH Audit					
PCMH NCQA. End 2018	26.0	54.0	83.0	75.3 (72.8–77.8)	6.2
PCMH NCQA. End 2021	26.0	66.0	81.0	72.8 (71.1–74.5)	4.2
PCMH NCQA. End 2022	26.0	77.0	88.0	83.5 (82.1–84.9)	3.5
Chronic Diseases Care Audit					
CDC audit. End 2022	26.0	30.0	90.3	73.3 (67.0–79.6)	15.6
Clinical Key Performance Indicators aggrigate percentage					
Clinical KPI. End 2021	26.0	50.5	73.2	64.9 (62.6–67.3)	5.8
Clinical KPI. End 2022	26.0	51.0	77.0	63.9 (61.3–66.7)	7.0
Clinical KPI. Mid 2023	26.0	57.3	84.3	72.9 (70.3–75.3)	6.4
Preventive Key Performance Indicators aggrigate percentage					
Preventive KPI. End 2022	26.0	33.0	58.0	47.8 (45–50.6)	7.0

In 2021, the best-performing center regarding PCMH implementation based on the NCQA PCMH score audit was achieving 83.5% of the best possible score (81 points out of a possible 97), while the worst-performing was 68% (66 out of 97). In 2022, with more efforts toward recovery from the pandemic, the best performing was 90.7% (88 out of 97), and the worst was 79.4% (77 out of 97). A clear improvement was observed in 2022 following very difficult years of AHS services directed toward COVID-19 prevention and mitigation. Compared to the first NCQA assessment done in AHS in 2018 based on the 2017 standards, the best that could be achieved in AHS was 83 points (92.2%), and the worst was 54 (58.7%). In 2022, adherence to structured care in the management of chronic diseases was 73.3% on average, with top-performing centers achieving 90%. Achievement of the best targets in clinical and preventive KPI for the year 2022 was 77% and 58%, respectively. The clinical KPI showed persistent improvement from 2021, from 73.2% in 2021 to 77% in 2022 to 84.3% in 2023 (Table 1).

### 3.3 PCMH and CDC scores in relation to KPI and utilization

**Figure 1:**
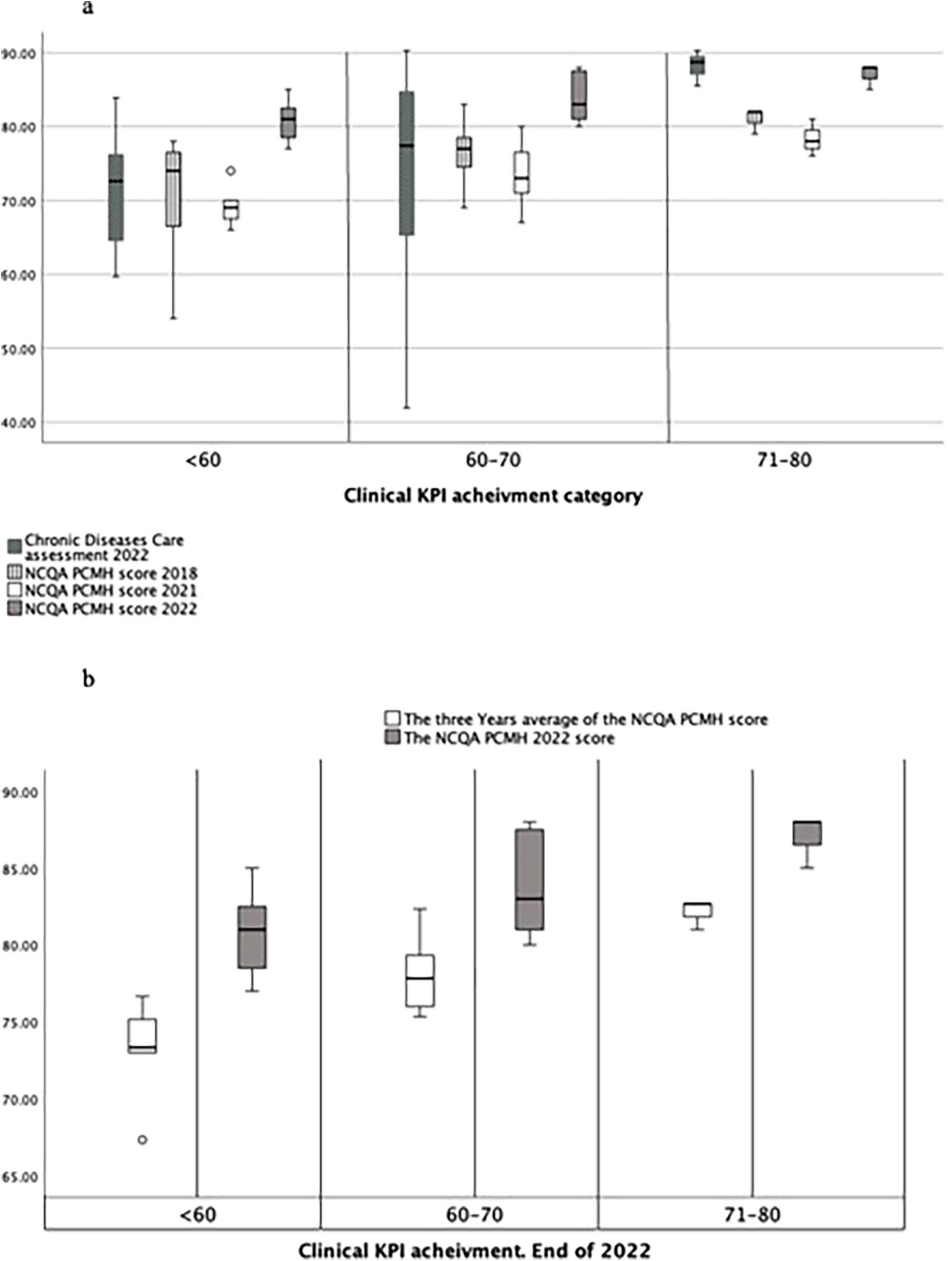
**(a)** NCQA PCMH assessments' scores over the years 2018, 2021, and 2022 and the chronic disease management program standards implementation related to three clinical KPI achievements categories: low, less than 60, moderate, 60 to 70, and higher performers, above 70. **(b)** Centers' adherence to the NCQA PCMH standards (average of the three years 2018, 2021, and 2022 NCQA PCMH scores) compared to 2022 NCQA PCMH performance.

Figure 1 shows the NCQA PCMH assessment scores for 2018, 2021, and 2022 and the implementation of chronic disease management program standards related to three clinical KPI achievement categories: low (<60), moderate (60–70), and high (>70) performers.

It shows that the best-performing centers, based on KPI achievement, demonstrated better implementation of the PCMH system according to the NCQA assessment. Figure 1b shows that centers with longer adherence to the NCQA PCMH standards (average of 2018, 2021, and 2022) were enabled to better meet the clinical KPIs. Centers that achieved better adherence in 2022 to NCQA PCMH standards, compared with 2018 and 2021, achieved less in clinical KPI than those that were better in the 3 years. Figure 1a shows that the score of the NCQA PCMH assessment before the COVID-19 pandemic was better than that of 2021, 2.5% decrease, indicating the negative impact of the pandemic on quality of care, which was followed by an improvement in the recovery year of 2022 with centers showing high implementation of NCQA PCMH and CDC standards. Nevertheless, in terms of the 2022 clinical KPI, all the better-performing centers were superior to others in adherence to these standards.

**Figure 2:**
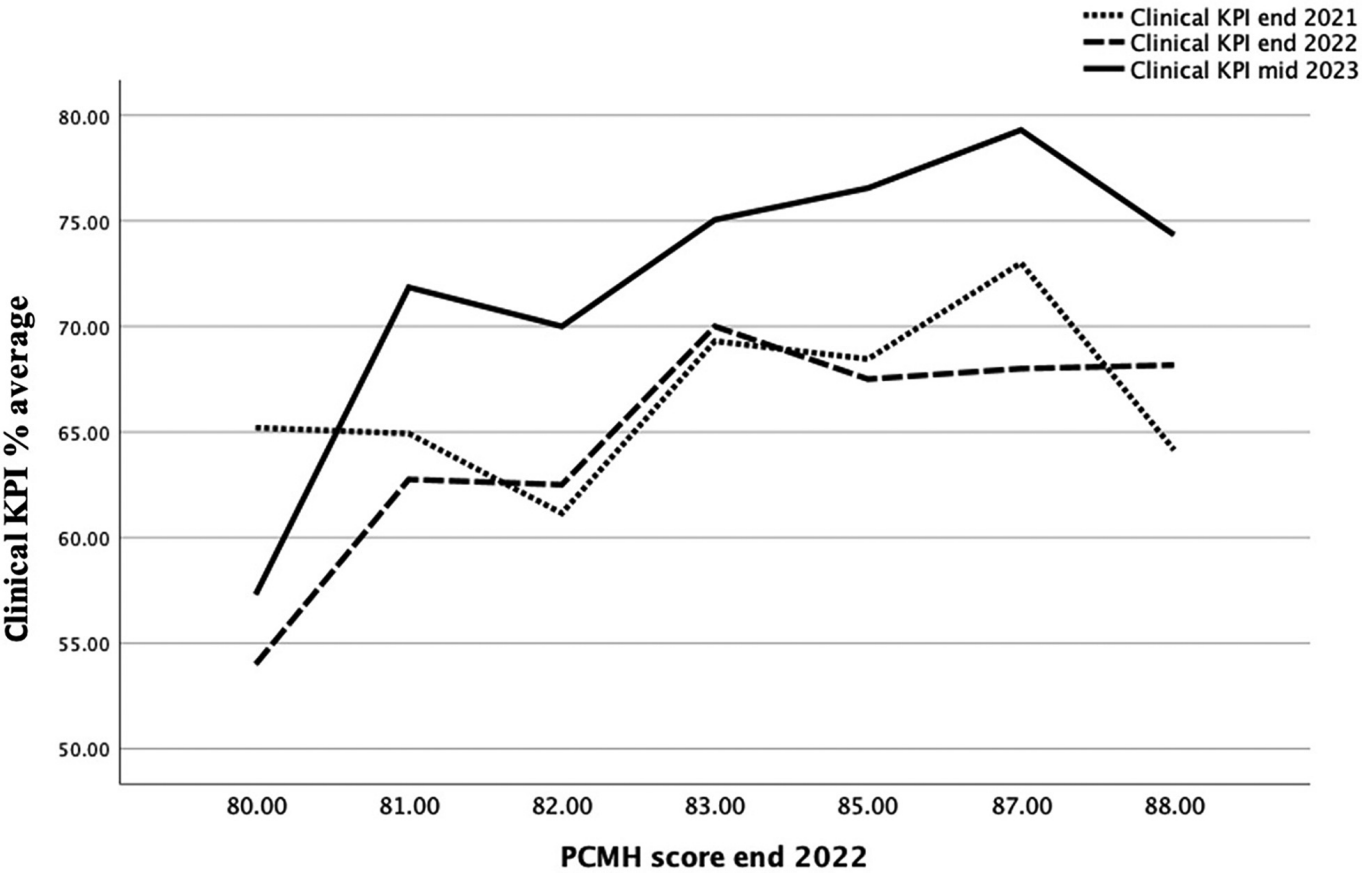
Clinical KPI average of all centers in relation to NCQA PCMH scores at the end of 2021, end of 2022, and mid-2023.

Linear regression showed that better implementation of the NCQA PCMH and CDC programs was associated with better KPI outcomes and not related to changes in centers' financial revenue. In the 2023 mid-year clinical KPI, care was superior in centers with better NCQA PCMH scores at the end of 2022, except in rural centers (*B* = 0.479, *p* = 0.044). However, rural centers demonstrated a similar association between the implementation of PCMH and clinical KPI achievement in the years before (Figure 2).

A significant association (*B* = 0.447, *p* = 0.03) was observed between the end-of-2022 clinical KPI performance of all centers and the PCMH NCQA end-of-year 2022 score using linear regression. However, centers' financial revenue, reflecting the financial implication, was not significantly associated with a better end-of-year clinical KPI (*B* = 0.209, *p* = 0.29). Similarly, through univariate linear regression, a higher CDC program assessment at the end of 2022 was significantly associated with centers that better performed in clinical KPI (*B* = 0.480, *p* = 0.013). The Chronic Diseases Program assessment at the end of 2022 was positively and significantly associated with the higher performance of centers regarding the NCQA PCMH standards (*B* = 0.647, *p* < 0.001). Linear mixed-effects model was used to account for center-level clustering, and the significant association was confirmed.

### 3.4 Correlation analysis of PCMH and CDC scores and outcome assessed (KPI and utilization)

The end of 2022 metrics (Table 2) analyzed by PCA emphasized the association between better outcomes in clinical KPI and adherence to NCQA PCMH and CDC standards. Significant correlations were observed between clinical KPI achievement in 2022 and the PCMH NCQA 2022 score (*p* = 0.004), preventive KPI achievement in 2022 (*p* = 0.028), higher centers' financial revenue (*p* = 0.038), and higher CDC program score in 2022 (*p* = 0.013). The PCMH NCQA 2022 score significantly correlated with a higher centers' financial revenue (*p* = 0.022) and a higher CDC program score in 2022 (*p* < 0.001). In addition, the CDC program score in 2022 significantly correlated with higher utilization, centers' financial revenue (*p* = 0.008), more lab orders (*p* = 0.017), and more prescriptions (*p* = 0.004). Preventive services significantly correlated with centers with better clinical KPI but not with any other variable.

**Table 2:** Correlations between the PCMH NCQA scores of the three years assessed, the volume of the services represented by the centers’ financial revenue, prescriptions, and radiological and laboratory requests, and the outcome KPI represented in the clinical and preventive KPIs in addition to chronic diseases care program assessment.

		NCQA assessment. End 2022	centers’ financial revenue 2022	Prescriptions number	Radiology orders	lab orders	Clinical KPI. End 2022	Preventive KPI. End 2022	NCQA assessment. End 2018	Chronic Diseases assessment 2022
NCQA assessment. End 2022	Pearson Correlation	1	0.447*	−0.127	0.316	0.084	0.541**	0.081	0.195	0.647**
	*P* value		**0**.**022**	0.535	0.116	0.684	**0**.**004**	0.695	0.34	**<**.**001**
centers’ financial revenue 2022	Pearson Correlation	0.447*	1	−0.561**	0.668**	0.136	0.409*	−0.094	−0.015	0.505**
	*P* value	**0**.**022**		**0**.**003**	**<**.**001**	0.509	**0**.**038**	0.647	0.943	**0**.**008**
Prescriptions number	Pearson Correlation	−0.127	−0.561**	1	−0.534**	−0.165	−0.266	0.059	0.028	0.546**
	*P* value	0.535	**0**.**003**		**0**.**005**	0.42	0.189	0.776	0.892	**0**.**004**
Radiology orders	Pearson Correlation	0.316	0.668**	−0.534**	1	0.0533**	0.222	−0.163	0.077	0.346
	*P* value	0.116	**<**.**001**	**0**.**005**		**0**.**005**	0.276	0.427	0.708	0.083
lab orders	Pearson Correlation	0.084	0.136	−0.165	0.533**	1	0.304	0.066	0.021	0.465*
	*P* value	0.684	0.509	0.42	**0**.**005**		0.131	0.749	0.918	**0**.**017**
Clinical KPI. End 2022	Pearson Correlation	.541**	.409*	−0.266	0.222	0.304	1	0.431*	0.235	0.480*
	*P* value	**0**.**004**	**0**.**038**	0.189	0.276	0.131		**0**.**028**	0.249	**0**.**013**
Preventive KPI. End 2022	Pearson Correlation	0.081	−0.094	0.059	−0.163	0.066	0.431*	1	−0.114	0.077
	*P* value	0.695	0.647	0.776	0.427	0.749	**0**.**028**		0.579	0.71
NCQA assessment. End 2018	Pearson Correlation	0.195	−0.015	0.028	0.077	0.021	0.235	−0.114	1	−0.019
	*P* value	0.34	0.943	0.892	0.708	0.918	0.249	0.579		0.927
Chronic Diseases assessment 2022	Pearson Correlation	0.647**	.0505**	.0546**	0.346	0.465*	0.480*	0.077	−0.019	1
	*P* value	**<0**.**001**	**0**.**008**	**0**.**004**	0.083	**0**.**017**	**0**.**013**	0.71	0.927	

Pearson correlation analysis (PCA).

*The correlation is significant at *p* < 0.05 level.

**The correlation is significant at *p* < 0.01 level.

Bold values indicates significant results.

## 4 Discussion

### 4.1 PCMH and CDC programs effectiveness

This paper reports 10 years of the real-world implementation of evidence-based standards, the NCQA PCMH, in an ambulatory care setting and over 18 years of a CDC management program in Abu Dhabi (12, 13). The positive association between PCMH recognition and clinical performance in healthcare centers was well established in quality of care, QoL, utilization, cost, and patient experience (21, 22, 31). However, this study supports the effectiveness of its implementation in Abu Dhabi and reports sustainability over nearly 10 years, with improvements in PCMH even in the less-performing centers.

This superior performance occurred without negatively impacting utilization, which is measured by patient volume, investigations, and prescription numbers, and is similar to US-based studies (32). In fact, there was an increase in labs and prescriptions, indicating that more scheduled, evidence-based care was implemented in accordance with standards and KPIs. For example, more patients were recalled for the population health program, prediabetes, early hypertension detection, CKD, dyslipidemia, and stroke if they did not have a scheduled appointment, and there were more radiology and labs as part of proactive planned care, such as panel management and coordination of population health programs, as well as medication adjustments to close care gaps. These results align with the implementation outcomes of PCMH in the US, as detailed in the NCQA PCMH evidence report (33).

### 4.2 Pandemic resilience

The superior care in centers with better implementation and adherence to standards was sustained during the pandemic, as shown by repeated assessments, indicating significant effectiveness and the value of the structure and processes put in place. This supports the reported importance of the preexisting structural health care system's status. A study of eight countries in Latin America and the Caribbean found that weaknesses in the system prior to COVID-19 were a significant factor in the extent of disruption to healthcare delivery caused by the pandemic. Additionally, the impact of postponed and forgone primary care and hospital services added to these challenges. All of these issues have called for innovative strategies to maintain and restore services, such as public-private financing and coordination, telemedicine, and new roles for primary care (34). An example within the US of implemented corrective action is the Veterans Health Administration (VHA) Preventive Health Inventory (PHI) program, a multicomponent care management intervention, including a clinical dashboard and templated electronic health record notes, to support primary care in delivering CDC and preventive care that the pandemic had delayed. A higher use of this multicomponent care management intervention was associated with improved quality-of-care metrics (35). All of these are core areas in PCMH standards, and explain the better performance of centers with higher PCMH scores. This study offers important perspectives by focusing on family medicine-based interventions, rather than administrative operational interventions, which have been frequently reported in COVID-19 healthcare services research (36).

### 4.3 Sustainability lessons

When it comes to sustainability and progress in PCMH implementation, recognition and maintenance of that recognition is standard practice in the United States. However, in the current context, the commitment made by management and teams in the healthcare centers has been longstanding. Therefore, comparing this sustainability to others is challenging, but it remains a crucial area for study, as the benefits and return on investment could have played a role in fostering such commitment.

A strength in this reported experience is that sustainability was achieved despite the lack of recognition, similar to the US system. A US study concluded that small and medium-sized practices may experience difficulty with the financial burden of PCMH recognition, and transformation is disruptive to practices, requiring the commitment of leadership and personnel. However, their value requires policies that recognize and meet the requirements of on-site practice leaders to promote primary care practice transformation (37–39). Thus, any transformation toward PCMH is recommended, although the value of certification and recognition cannot be determined from this study. In AHS, the sustainable leadership decision to support its implementation demonstrates the healthcare team's commitment to adhering to standards, even during the pandemic. Notably, the family medicine-based care (40) can never be achieved without the PCP. The link to the patients in their panel is the center of all elements in the programs described. In AHS, empanelment is facilitated through the EMR, and reports and care coordination are easier with a PCP identified for each patient, and each PCP has their own panel.

A unique sustainability attribute in this country's context is that practice improvement is centralized and governed in coordination with key departments to facilitate and control its quality and implementation. Davy et al. supported this, emphasizing the commitment of leaders and spreading awareness (16). Ultimately, this study highlights the clear processes and outcomes that have been followed over time. In many studies, sustainability lacks an explicit definition of outcome variables; therefore, research cannot accumulate or disconfirm findings on sustainability predictors (5).

### 4.4 Experience has unique characteristics

The experience has unique characteristics worth considering for potential application elsewhere. First, it describes a mixed intervention implementation with individual elements of the standards whose uptake is influenced by the distinctiveness of the centers and their team efforts. The level of PCMH implementation seemed to be a determining factor of the team efforts, as concluded by (23). Advanced PCMH practices emphasized changes in the continuity of care, highlighting a focus on personal relationships rather than systemic change. Here, all the centers were on a shared journey as a network; however, analyzing the variability of efforts and their determinants could serve as a valuable research interest.

Second, while the PCMH standards are comprehensive and encompass numerous family medicine principles, the implementation of CDC programs in AHS since 2004 has been beneficial. The CDC program has been more prescriptive in structuring the flow of patients' and teams' responsibilities. Through the EMR, which serves as the foundation for population health programs, it connects to a vast pool of the population from which patients with chronic diseases are continuously identified. This could help improve adherence, as established and agreed-upon pathways and workflows can enhance the uptake of program strategies.

Rural centers did not differ from other centers in terms of the association between high PCMH scores and clinical KPI achievements. However, in 2023, there was an indication of a difference, with no association observed. More data regarding PCMH implementation in 2023 in rural centers can explain this finding. Gale et al. stated that although the readiness of the rural center for PCMH implementation varied, rural centers performed best on some NCQA PCMH standards, but many were challenging (41). Similarly, in Abu Dhabi, there was no difference in resources and infrastructure. However, owing to the low populations in certain regions, the teams were smaller, and care coordinators were not available in certain centers and were shared in others. Furthermore, the population demographics differed, and this affected the clinical KPI performance of the rural centers. This finding highlights the importance of this discipline in rural settings, suggesting proper staffing, such as shared care coordinators between centers.

This experience reporting has a limitation similar to those in healthcare services research: the difficulty of attributing effectiveness to a specific implemented element. Which were the most impactful elements? This was a challenge in studies in the US, where no PCMH implementation resembled others. Bodenheimer (42) noted that “because PCMH is a diffuse collection of initiatives rather than a focused intervention, evaluation is difficult” (42). Flieger (43) stated, “If you have seen one medical home, you have seen one medical home” (43). Research in this area could be more easily conducted in the financial domain, as posited by Burton et al. (20), who identified components of the PCMH model of care associated with lower spending and utilization among Medicare beneficiaries (20). However, identifying the same for the quality of care and patient experiences may not be easy as they are not as easy to quantify. This is more complex in unique settings, such as Abu Dhabi, as the confounders differ, and it is the only one in the region; therefore, generalizability is a risk. Nevertheless, the transferability of the core elements of the standards and the success of implementation, sustainability over time, and positive association with key clinical outcomes encourage the better delivery of family medicine services.

### 4.5 Identified challenges and opportunities for future improvements

The improvement in patient care associated with better adherent centers has reached international benchmarking levels in certain recognition programs, such as diabetes, stroke, and heart disease (44, 45). However, inertia was observed, probably attributed to factors that can only be confirmed by studies. The open-access system is allowing patients to break their continuity with their PCP, and care is interrupted by many options from different providers, which interfere with the care planned in CDC clinics. This open-access system facilitates timely accessible care; however, that is to be weighed against the duplication of services and less adherence to appointments and PCP continuity, with patients visiting urgent-care clinics for routine chronic care. The lack of evidence on reducing visits in this study may be linked to the fee-for-service system. No association was observed between the PCMH score and financial outcome, even when controlling for rural centers. This variability needs to be studied to determine its impact on determinants of health-seeking behavior and physician practice. In the US, where the fee for services is highly implemented, there is migration out of the system. This is due to the high-cost, low-value outcomes and increased interest in initiatives in primary care, particularly PCMH, which have shown better savings in centers implementing PCMH (46). Friedberg et al. (47) observed that practicing physicians find it challenging to keep up with the proliferation of models, and payment models have become increasingly complex since 2014, prompting practices to invest in understanding these complex alternative payment systems. Furthermore, they discovered that risk aversion was prominent among physician practices (47). Neumann (48) used Medical Expenditure Panel Survey data to analyze the relationship between primary care spending, PCMH implementation score, and health outcomes in 29 states and discovered that greater primary care investment led to better outcomes. Furthermore, PCMHs can help to reduce costs and improve population health (48).

The challenges of fulfilling the core functions of primary care, first contact, continuity, coordination, and comprehensiveness are within the confines of the fee-for-service (49). However, with the present PCMH-based structure and processes and CDC team in all centers, as well as informational continuity and care coordinators completing tasks, the center teams maintained continuity to achieve results.

### 4.6 Strengths and limitations

This study, which involved a relatively large network of primary healthcare centers with a long period of follow-up, demonstrated the value of investments in PCMH standards, with a focus on structured care in CCM-based disease management programs for chronic diseases, resulting in improved quality of care and utilization. Limitations that are important to acknowledge include the fact that data on average per-patient total spending or costs were not available, with only direct service charges being used. Additionally, the Abu Dhabi healthcare system provides free insurance for all UAE nationals, who make up the majority of AHS patients, although a fee is charged for the services. Both factors restrict the conclusions regarding financial impact and only suggest a lack of revenue loss, highlighting the need for further focused research.

## 5 Conclusion

The Abu Dhabi AHS investment and success in implementing evidence-based interventions demonstrated good-to-excellent implementation of both programs. Excellent sustainability was evident over the years, despite the noticeable decrease in PCMH scores and KPIs during the COVID-19 pandemic, which was followed by the highest score since the program began. The setting and strategies discussed underscore significant issues for sustainability research and provide evidence of the importance of maintaining interventions over time.

## Data Availability

The datasets presented in this article are not readily available because It is institutional administrative data. Requests to access the datasets should be directed to latifa.mohammad@gmail.com.
